# Phage transcription activator RinA regulates *Staphylococcus aureus* virulence by governing *sarA* expression

**DOI:** 10.1007/s13258-022-01352-8

**Published:** 2022-12-15

**Authors:** Ming Jiang, Yilin Li, Baolin Sun, Shiwen Xu, Ting Pan, Yujie Li

**Affiliations:** 1 College of Life Science and Technology, Mudanjiang Normal University, Mudanjiang, 157012 Heilongjiang People’s Republic of China; 2 Department of Life Science and Medicine, University of Science and Technology of China, Hefei, 230026 Anhui People’s Republic of China

**Keywords:** *Staphylococcus aureus*, RinA, SarA, Virulence

## Abstract

**Background:**

*Staphylococcus aureus* is a major human pathogen, that can lead to various community- and hospital-acquired infections. RinA is a transcription activator of *S. aureus* phage φ 11 involved in phage packaging and virulence gene transfer. However, little is known about the molecular mechanism of RinA in the regulation of virulence.

**Objective:**

We aimed to explore a novel contribution of RinA in the regulation of virulence and provide a new drug target in the treatment of *S. aureus* infections.

**Methods:**

The specific functions of RinA in *S. aureus* were analyzed by the methods of growth curve, real-time quantitative PCR (RT-qPCR), subcellular localization, electrophoretic mobility shift assay (EMSA), infection model of *Galleria mellonella* larvae and the mouse subcutaneous abscess model.

**Results:**

In this study, we demonstrated that RinA is a protein evenly distributed in the cytoplasm of *S. aureus*, and its deletion could cause the growth defects. RT-qPCR and EMSA determined that *rinA* could negatively regulate the expression of *sarA* by directly binding to its promoter, and vice versa. The *Galleria mellonella* larvae infection and mouse subcutaneous abscess models revealed that the *rinA* mutant strain exhibited obvious virulence defects. When *sarA* is knocked out, the virulence of *S.aureus* had no significantly changes whether *rinA* is knocked out or not.

**Conclusion:**

Our fndings demonstrated that phage transcription activator RinA regulates *S. aureus* virulence by governing *sarA* expression.

## Introduction

*S. aureus* is a major human pathogen that causes multiple diseases such as skin infections, bacteremia, pneumonia, endocarditis, and toxic shock (Lee et al. 2018). Furthermore, owing to their adaptability to different environmental conditions, hospital- and community-acquired infections are surging (Dayan et al. 2016). The main treatment for *S. aureus* relies heavily on the use of antibiotics. However, the emergence of drug-resistant strain brought a more serious burden for the treatment of *S. aureus* infections (Lakhundi and Zhang 2018). *S. aureus* can produce an arsenal of virulence factors, such as adhesion proteins, superantigens, pore-forming toxins, ADP-ribosylating toxins and proteases. Moreover, multiple complex virulence regulatory systems including the quorum-sensing system, two-component system and the SarA protein family, have evolved. These virulence factors and virulence regulatory systems together contribute to the high pathogenicity of *S. aureus* (Bronner et al. 2004; Jenul and Horswill 2019).

The SarA protein family plays a crucial role in regulating the virulence of *S. aureus* (Cheung et al. 2008). The staphylococcal accessory regulator locus (*sarA*) encodes a DNA-binding protein that regulates the expression of more than 100 genes (Sterba et al. 2003). SarA can indirectly regulate the expression of virulence genes, including α-hemolysin-, protein A-, and fibronectin-binding proteins in an *agr*-dependent manner, furthermore, it can directly bind to the promoter regions of virulence genes to up-regulate or down-regulate their expressions (Chien Y et al. 1999; Pan and Simon 1998; Yueh-tyng Chien and Cheung 1998). Additionally, the mutation of *sarA* could decrease biofilm formation and prevent the accumulation of extracellular toxins (Zielinska et al. 2012).

The RinA family contains two proteins, RinA and RinB, RinA is basic DNA-binding protein that directly activates gene expression, while RinB is acidic and does not appear to regulate gene expression, however, RinB may play a minor role in activation such as providing stability to RinA (Ferrer et al. 2011; Ye and Lee 1993). The late operons of many phages control the morphogenetic and lysis genes of *S. aureus*, previous studies have shown that the RinA family of phage-encoded proteins are activators required for the transcription of late operon in a crowd of temperate staphylococcal phages (Ferrer et al. 2011; Quiles-Puchalt et al. 2013). To control the expression of the late operon, the RinA protein binds to a DNA repeat region situated upstream of the terminase small subunit gene (*ter*S) (Quiles-Puchalt et al. 2014). Moreover, the deletion of *rinA* abolishes the formation of functional phage particles and significantly reduces the transfer of phage and virulence factors encoded by the pathogenicity islands (Ferrer et al. 2011). The control of phage-mediated transfer of virulence factor is a conserved mechanism that regulates horizontal gene transfer (HGT), which significantly impacts the evolution of bacterial pathogens between microorganisms (Olszak et al. 2017). Phages are one of the types of *S.aureus* Mobile genetic elements (MGEs) which can impact expression of virulence determinants by either positive or negative lysogenic conversion. (Malachowa and DeLeo 2010). In horizontal gene transfer between bacteria, phages play an essential role by acting as gene transfer vectors and carrying virulence factors, these two functions can be observed in *S. aureus* phages (McCarthy et al. 2012). Although RinA acts upon the transfer of virulence factors, its specific impact on the *S. aureus* pathogenicity is unknown. Moreover, whether the regulation of RinA in *S. aureus* virulence depends on *sarA* expression also needs to be uncovered.

Herein, it was demonstrated that RinA could negatively regulate the expression of *sarA* by directly binding to its promoter region, and vice versa. Additionally, the contribution of RinA to the pathogenicity of *S. aureus* was further confirmed by using the infective *G. mellonella* larvae and the mouse subcutaneous abscess model. In summary, the results revealed that RinA positively regulates the virulence of *S. aureus* in a SarA dependent manner. These insights on the underlying molecular mechanisms can guide the development of novel anti-infective strategies.

## Materials and methods

### Bacterial strains, plasmids, and growth conditions

The bacterial strains and plasmids used in this study are listed in Table 1*. Escherichia coli* was grown (220 rpm) in a lysogeny broth (LB) medium or on lysogeny broth agar (LA), while *S. aureus* was grown (220 rpm) in a tryptic soy broth (TSB) medium or on tryptic soy agar (TSA) at 37℃. The appropriate antibiotics were used for plasmid selection and maintenance. They were added to the bacterial cultures at the following concentrations: 150 μg/mL ampicillin sodium salt or 50 μg/mL kanamycin sulfate for *E. coli* or 15 μg/mL chloromycetin for *S. aureus*.

**Table 1:** Strains and plasmids used in this study

Strain and plasmid	Relevant genotype^a^	Source^b^
*S.aureus* strains		
RN4220	8325–4 r^−^, initial recipient for modification of plasmids which are introduced into *S. aureus* from *E. coli*	NARSA
NCTC8325	HA-MSSA, wild-type	NARSA
Δ*rinA*	NCTC8325 strain deletion of *rinA*	This study
Δ*rinA::rinA*	*rinA* chromosomal complementationation of Δ*rinA*	This study
Δ*sarA*	NCTC8325 strain deletion of *sarA*	This study
Δ*sarAΔrinA*	NCTC8325 *sarA rinA* double mutant	This study
*E.coli* strains		
Trans5α	Clone host strain	TransGen
BL21(DE3)	Expression strain	TransGen
Plasmids		
pBTs	Shuttle vector, temperature sensitive, Amp^r^ Cm^r^	(Hu et al. 2015)
pBTs-Δ*rinA*	pBTs containing upstream and downstream fragments of *rinA*, for *rinA* deletion, Amp^r^ Cm^r^	This study
pBTs-Δ*sarA*	pBTs containing upstream and downstream fragments of sarA, for *sarA* deletion, Amp^r^ Cm^r^	This study
pET28 ( +)	Expression vector with a hexa-histidine tag, Kan^r^	Novagen
pET-*rinA*	pET28a( +) derivative, with ORF of *rinA*, Kan^r^	This study
pALC	Fluorescent vector with GFP	Novagen
pALC-RinA	pALC derivative, with ORF of *rinA* located at the C-terminal of eGFP, Amp^r^	This study

^a^*r*   restriction system negative, *Kan*^*r*^ kanamycin resistant, *Amp*^*r*^ ampicillin resistant, *Cm*^*r*^ chloramphenicol resistant

^b^*NARSA* Network on Antimicrobial Resistance in *Staphylococcus aureus*

### Construction of *S. aureus* mutant strains

Plasmid pBTs were utilized to construct the *rinA* mutant strain. The upstream and downstream regions of *rinA* from the genomic DNA of *S. aureus* NCTC8325 were amplified with primers *rinA*-LB-F/R and *rinA*-RB-F/R. Next, the products were connected by overlap extension PCR. The resulting fragments were digested with *Kpn*I and *Sal*I and then cloned into pBTs. Afterward, the resulting plasmid pBTsΔ*rinA* was electroporated into *S. aureus* strain RN4220 for modification and then transformed into *S. aureus* strain NCTC8325. An allelic replacement mutant was selected using a previously described method (Bae and Schneewind 2006) and was further confirmed by PCR and sequencing. The *sarA* mutant strains and *sarA rinA* double mutant strains were constructed in the same way. The *sarA* mutant and *rinA sarA* double mutant strains were constructed using a similar strategy by introducing the plasmid pBTsΔ*sarA* into the wild-type (WT) and *rinA* mutant strains, respectively. All primers used in this study are listed in Table 2.

**Table 2:** Oligonucleotide primers used in this study

Primer	Sequence (5′–3′)^a^	Application
*rinA-LB*-F(*Kpn*I)	GCGGAATTCGAGCTCggtaccGGCTACATTGAGTGTTGTAC	*rinA* deletion
*rinA-LB*-R	**AAAAACTGCCACTTTACTGCCAATT**GAATACCCTCCGTACAAATA	*rinA* deletion
*rinA*-R*B*-F	**AAACATATTTGTACGGAGGGTATTC**AATTGGCAGTAAAGTGGCAG	*rinA* deletion
*rinA*-R*B*-R(*Sal*I)	CTTGCATGCCTGCAGgtcgacGCCACAGTATACGCCTAGGA	*rinA* deletion
C-*rinA*-RB-F	TATACGTTCAATTGCGCTCTTAAACTCAAGGATTTTACCTCTTC	*rinA* complementationation
C-*rinA*-LB-R	TTTAAGAGCGCAATTGAACGTATAATCAACACATCAAGCAGGAA	*rinA* complementationation
*sarA-*LB-F(*Kpn*I)	GCGGAATTCGAGCTCggtaccGCGTTGATTTGGGTAGTATG	*sarA* deletion
*sarA*-LB-R	**CTTCACCAAATTGCGCTAAACAAAAG**TTTAAAACCTCCCTATTTG	*sarA* deletion
*sarA*-RB-F	**TGCATCAAATAGGGAGGTTTTAAAC**TTTTGTTTAGCGCAATTTGG	*sarA* deletion
*sarA*-RB-R(*Sal*I)	CTTGCATGCCTGCAGgtcgacGCAACATCAACTAGCATCAT	*sarA* deletion
RT-*pta*-F	AAAGCGCCAGGTGCTAAATTAC	RT-qPCR
RT-*pta*-R	CTGGACCAACTGCATCATATCC	RT-qPCR
*RT-rinA*-F	TAATGACACCGTGGATTCC	RT-qPCR
*RT-rinA*-R	CGTTCTTTCCTGCTTGATG	RT-qPCR
RT-*asgA*-F	CGTATCAGTTAAATGCGAGA	RT-qPCR
RT-*asgA*-R	GACACCTGATTGACCACTA	RT-qPCR
RT-*relA*-F	ACACTGGGCTTACAAAGAA	RT-qPCR
RT-*relA*-R	TGGCAACTCAATAACATCAC	RT-qPCR
RT-*ccpA*-F	GCTCGTGGACTTGAAGATA	RT-qPCR
RT-*ccpA*-R	CTACTACAGGTACAGATGATTG	RT-qPCR
RT-*srrA*-F	GATGGTATCCAGGTGGCA	RT-qPCR
RT-*srrA*-R	ACAGTTGTAGATTGCGTTCT	RT-qPCR
RT*-sarA*-F	ATGGTCACTTATGCTGACAA	RT-qPCR
RT-*sarA*-R	GGTTGTTTGTAGTTTAAATG	RT-qPCR
RT-*RNAIII*-F	GCCATCCCAACTTAATAACC	RT-qPCR
RT-*RNAIII*-R	ACGATAGCTTACATGCTAGA	RT-qPCR
RT-*saeR*-F	CGCCTTAACTTTAGGTGCAG	RT-qPCR
RT-*saeR*-R	ATAGGGACTTCGTGACCATT	RT-qPCR
RT-*sigB*-F	GGTGCCATAAATAGATTCGA	RT-qPCR
RT-*sigB*-R	CACCGATTACAGTAGGTACT	RT-qPCR
RT-*agrA*-F	CGTGGCAGTAATTCAGTGTA	RT-qPCR
RT-*agrA*-R	TATGGCGATTGACGACAAAG	RT-qPCR
RT-*agrC*-F	CTCGGATGAAGCTAAAGTAA	RT-qPCR
RT-*agrC*-R	AATCATGACGGAACTTGCGC	RT-qPCR
RT-*hla*-F	CCCGGTATATGGCAATCAAC	RT-qPCR
RT-*hla*-R	GGTAGTCATCACGAACTCGT	RT-qPCR
S10-F	AAACGACGGCCAGTGAATTCCCGTTCTTATGACTAATTAT	*rinA* location
S10-R	CCCTCCGTACAAATATGTTTAATCTCTTATTCGTCTACATTTAGT	*rinA* location
L-*rinA*-F(*BamH*I)	CGCggatccAGATTAAACATATTTGTACGGAGGG	*rinA* location
L-*rinA*-R	ACCAGAACCACCACCAGAACCACCGTCTTCCCCTAACTCTTCCG	*rinA* location
eGFP-F	GGTGGTTCTGGTGGTGGTTCTGGTGTCGCCACCATGGTGAGCAA	*rinA* location
eGFP-R	AAAAGCTTGCATGCCTGCAGCGAAAGTCCACCTCCTAAAATTGTCTATCAGGACTTGTACAGCTCGT	*rinA* location
E-*rinA*-F(*EcoR*I)	ATGGGTCGCGGATCCgaattcATGACTAAAAAGAAATACGGATTAAAATTATCAACAGTTCGAAAGTTAG	*rinA* expression
E-*rinA*-R(*EcoR*I)	TTGTCGACGGAGCTCgaattcTCAGTCTTCCCCTAACTCTTCCGCCAATCTAGATA	*rinA* expression
P*-rinA*-F	GTCGGAGTAGATGATGTGGT	EMSA
P*-rinA*-R	GAATACCCTCCGTACAAATA	EMSA
P-*sarA*-F	CTAAACCAAATGCTAACCCA	EMSA
P-*sarA*-R	GCCATGTTTAAAACCTCCCT	EMSA
P-*rinA*-FAM-R	GAATACCCTCCGTACAAATA	EMSA
P-*sarA*-F*AM*-R	GCCATGTTTAAAACCTCCCT	EMSA

^a^Lowercase letters indicate restriction sites. Letters in bold indicate complementationary sequences used for overlap pcr ligation

### Construction of the complementation strain

To construct the *rinA* chromosomal complementation, the DNA fragment of the full-length *rinA* ORF and the flank upstream and downstream regions were amplified from the genomic DNA of *S. aureus* NCTC8325 with primers C-*rinA*-LB-F/R and C-*rinA*-RB-F/R. Next, the resulting fragments were digested with restriction enzymes and then cloned into pBTs. The resulting plasmid pBTs-LB-*rinA*-RB was first electroporated into *S. aureus* strain RN4220 for modification, and then transformed into the *rinA* mutant strain. The allelic replacement complementation strain was selected using the same method described above and was further confifirmed by PCR and sequencing.

### Total RNA isolation, cDNA generation, and real-time quantitative reverse transcription-PCR

Overnight cultures of *S. aureus* were diluted to an OD_600_ of 0.05 in TSB, grown to the indicated cell density and collected. Afterward, the collected cells were processed with RNAiso plus (TaKaRa) and lysed with 0.1 mm-diameter-silica beads in the FastPrep-24 system (MP Biomedicals) to extract the total RNA. The PrimeScript 1st Strand cDNA synthesis kit (TaKaRa) was employed to synthesize cDNA. Then, RT-qPCR was performed with SYBR Ex Taq premix (TaKaRa) using the StepOne real-time PCR system (Applied Biosystems) to quantify the relative gene expression. *pta* was used as the internal reference gene to normalize the gene expression (Wang and Sun 2021).

### Growth curves

Overnight cultures of *S. aureus* were diluted to an OD_600_ of 0.02 in TSB, added into a 96-well plate, and grown at 37℃ with shaking (220 rpm). The optical density was measured at 600 nm every hour using a microplate reader (Elx800; Bio-Tek). When the bacteria reached the stationary phase (about 12 h), the measurement was stopped.

### Subcellular localization of RinA

To detect the cellular localization of RinA, the DNA fragment of the full-length *rinA* ORF was amplified using PCR, and RinA was fused with eGFP at its C-terminal. The fusion gene was controlled by *S. aureus* S10 promoter. The RinA::eGFP fluorescent expression strain was confirmed by PCR and sequencing. An inverted confocal laser scanning microscope (FV1000, Olympus) was utilized for observation. An argon ion laser (Ex = 488 nm, Em = 515–530 nm) was used to observe fluorescent signals of eGFP. Finally, the confocal images were captured using the FV10-ASW 4.2 Viewer software (Olympus).

### Expression and purification of RinA

The 6-His-tagged RinA was expressed and purified using standard procedures. The DNA fragment of the full-length *rinA* ORF was amplified from the genomic DNA of *S. aureus* NCTC8325 using primers P*rinA*-F/P*rinA*-R, cloned into the expression vector pET28a to obtain the plasmid pET28a-*rinA*, and transformed into *E. coli* BL21 (DE3). The transformant was grown in LB medium at 37 °C to an OD_600_ of 0.6–0.8 and induced with 0.5 mM isopropyl-β-D-1-thiogalactopyr-anoside (IPTG) at 16 °C for 12 h. Next, the cells were harvested, resuspended in a protein buffer (50 mM Tris–HCl, 300 mM NaCl, pH 8.0) and lysed by sonication on ice. An Ni–NTA agarose solution (Novagen) was used to purify the protein with His tag at the C-terminus. The bound protein was eluted with an elution buffer (50 mM Tris–HCl, 300 mM NaCl, 300 mM imidazole, pH 8.0). The imidazole in the eluate was removed using a protein buffer, and the purified proteins were stored at − 80 °C until use. SDS-PAGE analyzed the purity of the protein and the protein concentration was determined using the bicinchoninic acid (BCA) assay with bovine serum albumin as the standard.

### Electrophoretic mobility shift assay

The FAM-labeled DNA fragment containing the promoter region of the target gene was amplified from the genomic DNA of *S. aureus* strain NCTC8325. The unlabeled fragment of the promoter was added as specific competitors, while that of the *pta* ORF was added as non-specific competitors. The FAM-labeled probe, different concentrations of proteins, and the protein buffer (50 mM Tris–HCl, 300 mM NaCl, pH 8.0) were mixed in 10 μL and incubated for 30 min at room temperature. After incubation, the mixtures were electrophoresed in a 5% native polyacrylamide gel in 1 × Tris–borate-EDTA (TBE) buffer. Finally, the band shifts were observed under the multifunctional laser imager.

### Infection model of *G. mellonella* larvae

Overnight cultures of *S. aureus* were diluted to an OD_600_ of 0.05 in TSB, then the late-logarithmic phase (OD_600_ = 6) bacterial solution was collected and diluted with PBS. Next, 20 μL was injected into the middle of the second gastropod (10^7^ CFU/mL). Larvae injected with 20 μL PBS were used as the control group. The larvae were incubated at 37℃ in the dark and observed at 24 h intervals for a week. The larvae were considered dead when they repeatedly failed to respond to physical stimuli. The primary outcome for the infection model of *G. mellonella* larvae was the rapidity and extent of mortality of the larvae assessed by the log-rank test.

### Mouse model of subcutaneous abscess

Outbred, immunocompetent female BALB/c mice between 5 and 6 weeks old were purchased from Gem Pharmatech Technology Company and raised to 6–8 weeks old in a specific sterile laboratory. The hair on both sides of each mouse's back was shaved off with a razor. The overnight cultures of *S. aureus* were diluted to an OD_600_ of 0.05 in TSB. The late-logarithmic phase (OD_600_ = 6) bacterial solution was then collected, washed twice, and diluted in sterile PBS. Afterward, the mice were anesthetized with 1% sodium pentobarbital and inoculated using subcutaneous injections in the bilateral back flanks with 5 × 10^7^ *S. aureus* cells in 50 μL PBS. The areas of abscess formation and skin lesions were monitored at 24 h intervals for 7 days and the skin lesions’ sizes were calculated using the maximal length × width. Each skin lesion (7 days after infection) from the mouse was excised and ground with a grinding rod in 1 mL PBS in iron gauze until it was homogenized. The number of CFU recovered from each lesion was then counted by serial dilution and plated onto LA plates. Next, the skin lesions were placed in 4% PFA Fix Solution for histopathological analyses. Paraffin embedding and hematoxylin and eosin (H&E) staining were performed by the Wuhan Servicebio Technology.

### Ethics statement

The use and care of mice in the present study strictly followed the guidelines adopted by the Ministry of Health of the People's Republic of China in June 2004. The protocol was approved by the Institutional Animal Care and Use Committee of the University of Science and Technology of China (USTCACUC182301015).

### Statistics

Statistical analyses were performed with Student’s *t* test or Mantel-Cox test. All error bars depict the standard errors of the means. Differences with a *p* value of 0.05 or less were considered significant. Significance was defined as *p < 0.05; **p < 0.01; ***p < 0.001; ****p < 0.0001. All experiments were performed in triplicate. For insect survival analysis, Kaplan–Meier survival curve was generated and analyzed for statistical significance with GraphPad Prism 7.0. Statistical details for each experiment can be found in the Figure Legends.

## Results

### Construction of *rinA* mutant

**Fig. 1:**
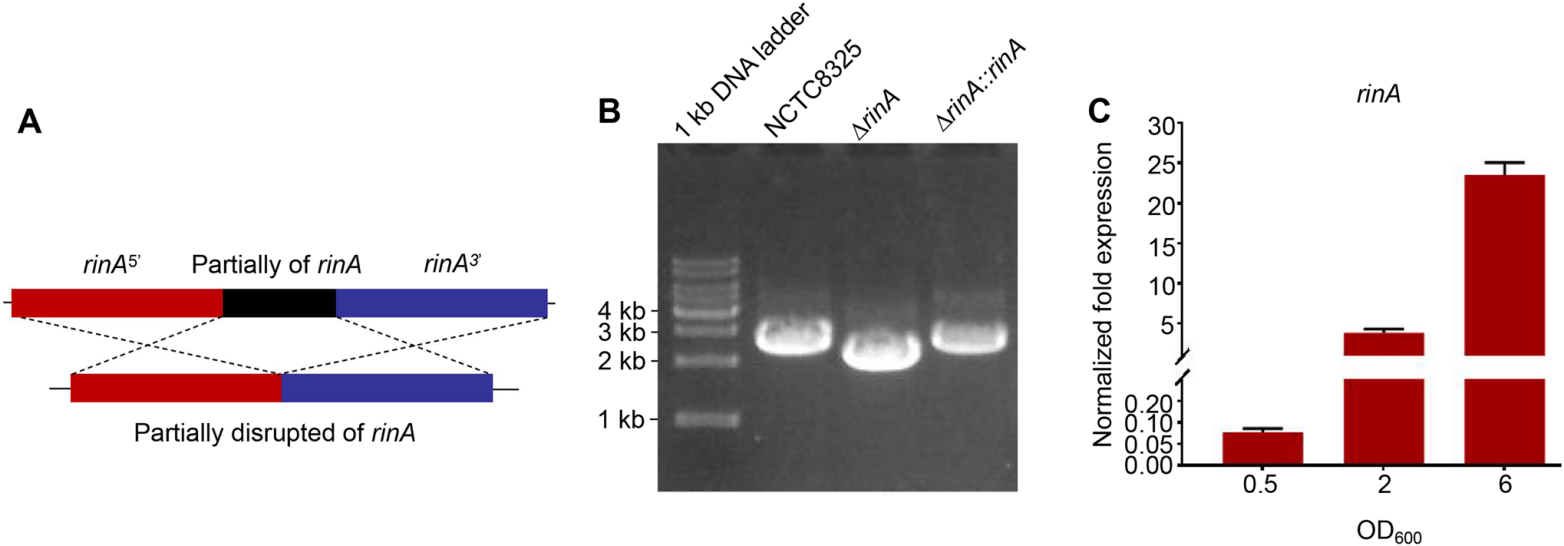
Construction of *rinA* mutant. **A** Diagram of *rinA* knockout strategy. **B** Evaluation of PCR products by agarose gel electrophoresis, from left to right were: 1 kb DNA Maker, wild-type, *rinA* mutant strain, *rinA* complementation strain. **C** The transcription level of *rinA* in different growth phases (OD_600_ = 0.5, 2, 6), the internal reference gene is *pta*

To explore the functions of RinA, the *rinA* disruption mutant was constructed by knocking out *rinA* from the genomic DNA of *S. aureus* NCTC8325 and the genome complementation strain was constructed by complementing *rinA* into the *rinA* disruption mutant (Figs. 1A, B). Moreover, the transcription levels of *rinA* during the different growth phases in the wild-type (WT) were detected by RT-qPCR. The results demonstrated that the transcription level of *rinA* was highest in the late-logarithmic phase and lower in the early-logarithmic and mid-logarithmic phase (Fig. 1C).

### Disruption of *rinA *renders growth defect of *S. aureus*

**Fig. 2:**
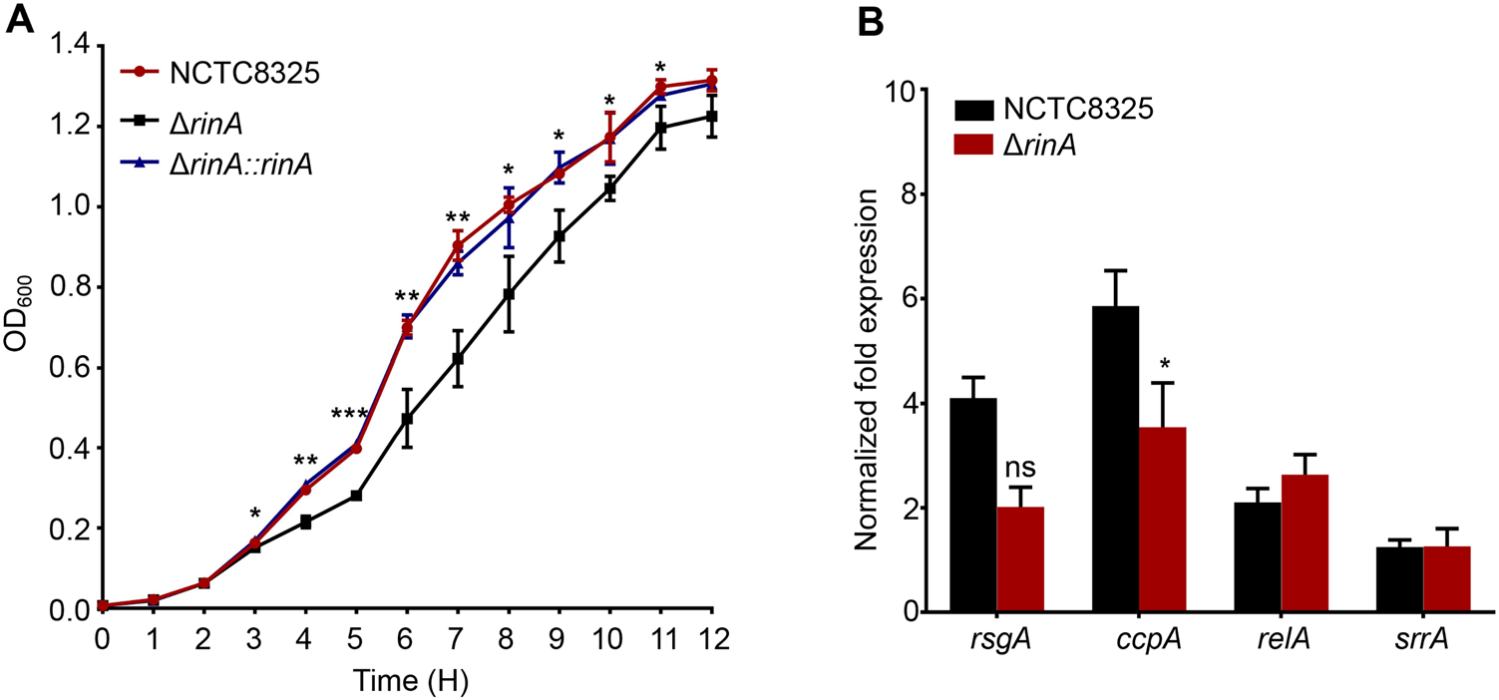
RinA deficiency slows down the growth rate of *S. aureus*. **A** The growth curve of the wild-type, *rinA* mutant and *rinA* complementation strains. The results were obtained from three independent experiments performed. Data is presented as the mean ± SD. Two-tailed Student’s *t*-test was used for the comparison of statistical significance. The wild-type strain was used as the reference. *p < 0.05; **p < 0.01; ***p < 0.001. **B** The transcription levels of genes related to growth in the wild-type and *rinA* mutant were evaluated through RT-qPCR. The means and standard deviations were then calculated. Data is presented as the mean ± SD. Two-tailed Student’s *t *test is used for the comparison of statistical significance. The wild-type strain was used as the reference. *p < 0.05

To determine the effects of RinA on the growth of *S. aureus*, the growth curve of the wild-type, *rinA* mutant and *rinA* complementation strains were measured*.* The results revealed that after 3 h, the growth rate of the *rinA* mutant strain was significantly weaker than the wild-type and *rinA* complementation strains (Fig. 2A). Since, *rsgA*, *ccpA*, *relA*, and *srrA* have been reported to affect the growth of *S. aureus* (Corrigan et al. 2016; Gentry et al. 2000; Pragman et al. 2007; Seidl et al. 2006), the transcription levels of these genes in the wild-type and *rinA* mutant strains were investigated. The results revealed that the transcription level of *ccpA* in the *rinA* mutant was significantly reduced compared to the wild-type (Fig. 2B). Taken together, the data suggested that RinA may be involved in the positive regulation of *S. aureus* growth in a *ccpA* dependent manner.

### Localization of RinA in *S. aureus*

**Fig. 3:**
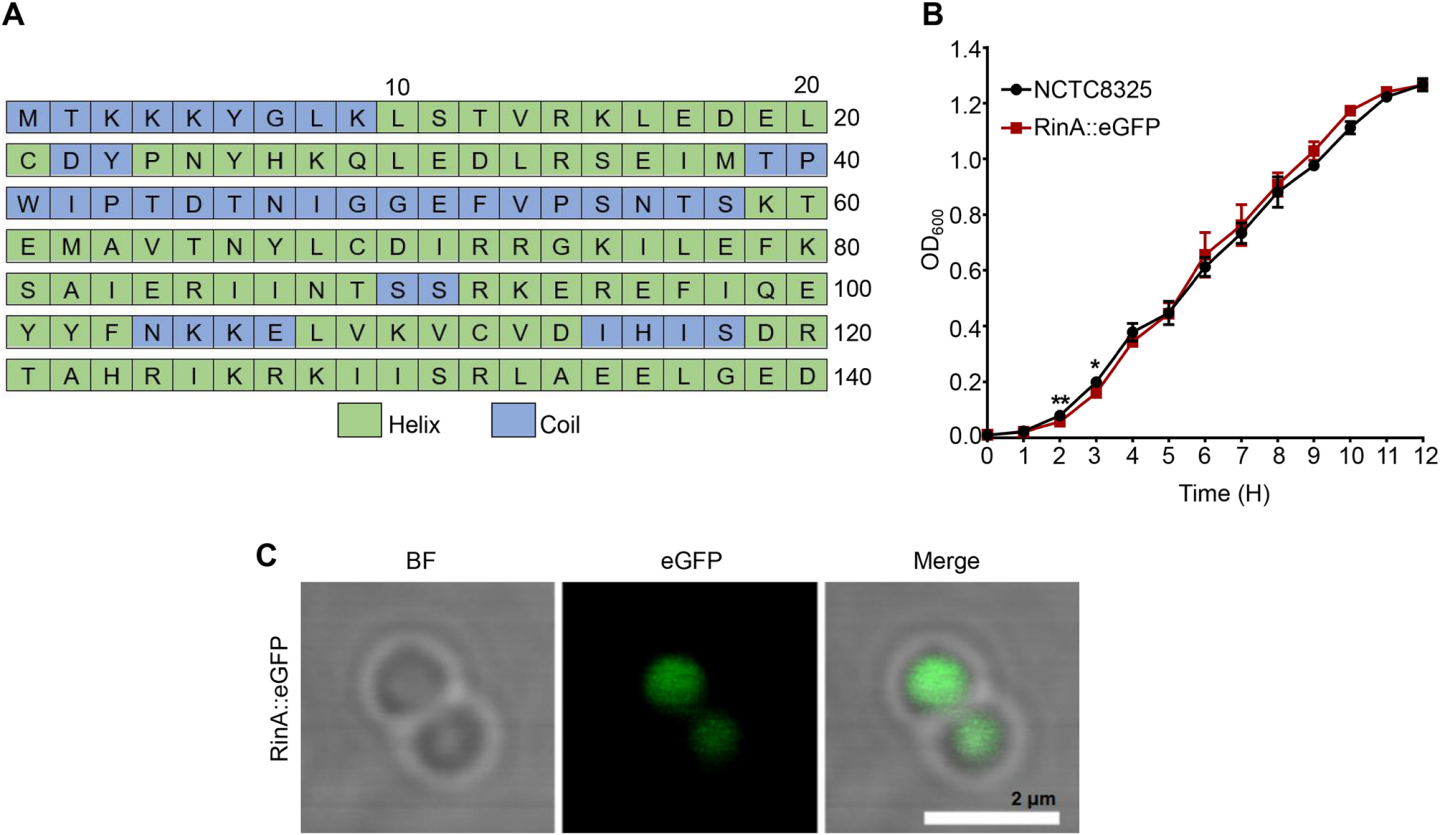
Localization of RinA in *S. aureus*. **A** PSIPRED online software predicted the predicted secondary structures of RinA. **B** The growth curve of the wild-type and RinA::eGFP fluorescent expression strain. The results were obtained from three independent experiments performed. Data is presented as the mean ± SD. Two-tailed Student’s *t* test was used for the comparison of statistical significance. The wild-type strain was used as the reference. *p < 0.05; **p < 0.01. **C** RinA::eGFP localized to the cytoplasm. The scale bar is 2 µm

The secondary structures of the RinA sequences were predicted using the PSIPRED online software (Buchan and Jones 2019) and the amino acid sequence analysis suggested that the secondary structures were mainly in the form of helixes (Fig. 3A). It has been reported that the localization of a protein in cells is closely related to its function. Therefore, the change of the subcellular location of a protein will affect its biological functions (Pishchany et al. 2009). To observe the localization of RinA, a RinA::eGFP fluorescent expression strain was constructed. Then, the growth curve was measured and the result showed that RinA::eGFP strain exhibited a similar phenotype to wild-type *S. aureus,* suggesting that fused with eGFP doesn’t impaire the function of RinA (Fig. 3B). The laser confocal microscope revealed that RinA was evenly distributed in the cytoplasm of *S. aureus* (Fig. 3C).

### RinA negatively regulates the expression of *sarA* by binding directly to the promoter and vice versa.

**Fig. 4:**
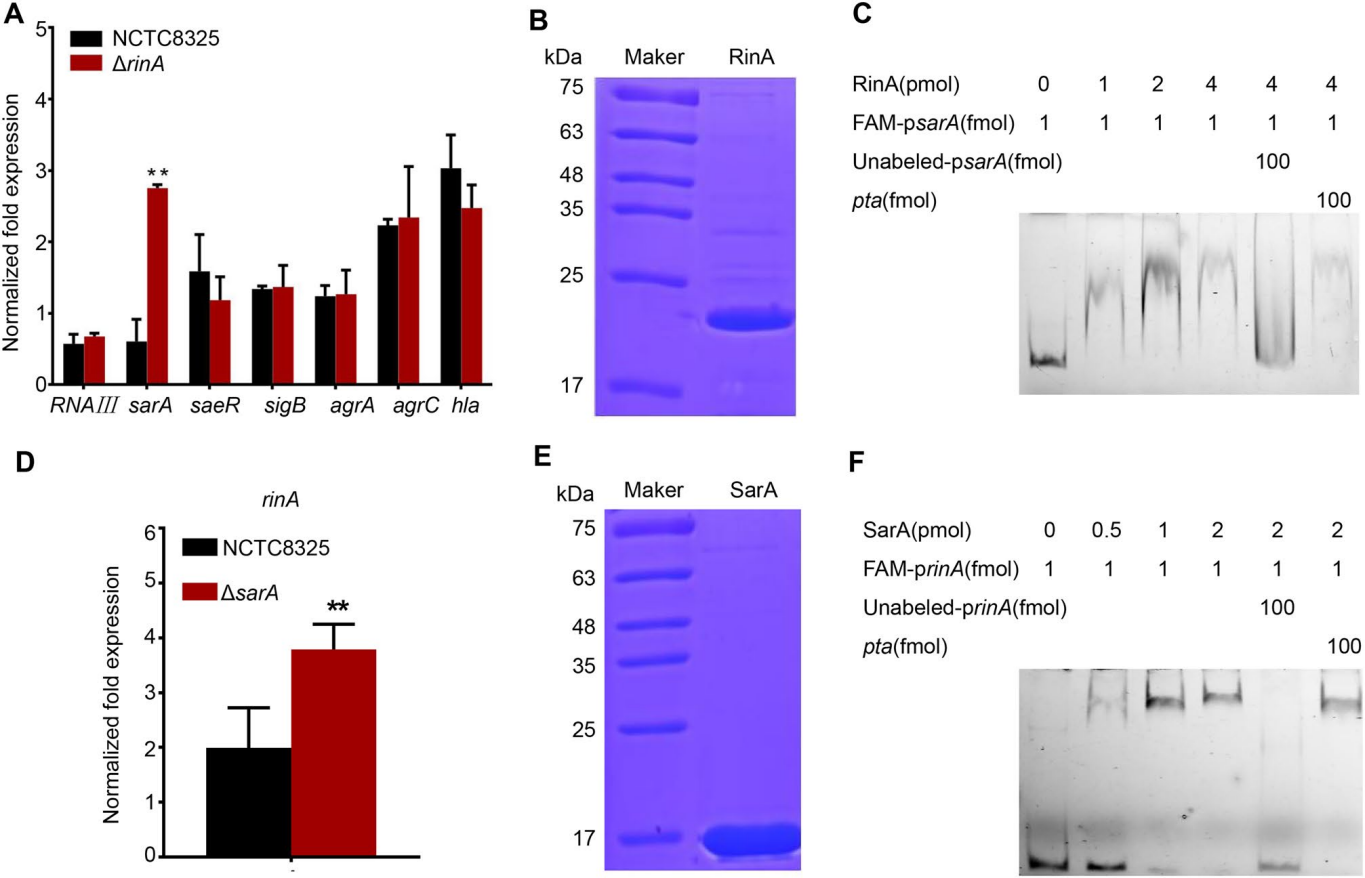
RinA negatively regulates the expression of *sarA* by binding directly to the promoter and vice versa. **A** The transcription levels of virulence genes in the wild-type and *rinA* mutant strains. The means and standard deviations were then calculated. Data is presented as the mean ± SD. Two-tailed Student’s *t* test is used for the comparison of statistical significance. The wild-type strain was used as the reference. **p < 0.01. **B** SDS-PAGE analysed of the purity RinA protein. **C** EMSA assay of the purified RinA with the FAM-labelled DNA fragments of *sarA*. Increasing concentrations of purified RinA and 1 fmol the FAM-labelled *sarA* probe were used in the reactions. The unlabelled probes were added as a specific competitor, and the unlabelled fragment of *pta* ORF region was added as a nonspecific competitor. **D **The transcription level of *rinA* in the late-logarithmic phase in the wild-type and *sarA* mutant strains. The means and standard deviations were then calculated. Data is presented as the mean ± SD. Two-tailed Student’s *t* test is used for the comparison of statistical significance. The wild-type strain was used as the reference. **p < 0.01. **E** SDS-PAGE analysed of the purity SarA protein. **F** EMSA assay of the purified SarA with the FAM-labelled DNA fragments of *rinA*. Increasing concentrations of purified SarA and 1 fmol the FAM-labelled *rinA* probe were used in the reactions. The unlabelled probes were added as a specific competitor, and the unlabelled fragment of *pta* ORF region was added as a nonspecific competitor

To investigate whether RinA was involved in regulating the virulence of *S. aureus*, the transcription levels of the virulence factors in the wild-type and *rinA* mutant strains were detected by RT-qPCR. The results determined that only the transcription level of *sarA* was significantly increased in the *rinA* mutant strain among all the genes detected. The transcription levels of other virulence factors such as *RNAIII*, *saeR*, *sigB*, *agrA*, *agrC* and *hla* had no significant changes (Fig. 4A). To that end, EMSA assay was utilized to further explore the regulatory mechanism. RinA was purified in vitro to obtain higher purity proteins (Fig. 4B). EMSA showed that RinA could directly bind to the *sarA* promoter to retard the migration of DNA probe, and as the protein concentration gradually increased, the phenomenon became more apparent. This binding could be disrupted using approximately 100-fold unlabeled *sarA* promoter fragment, while a 100-fold excess of unlabeled coding sequence fragment of *pta* did not have the same effect (Fig. 4C). To verify whether the regulatory function of RinA is affected by SarA, a *sarA* mutant strain was constructed, and the transcription level of *rinA* in *sarA* mutant was detected by RT-qPCR. The results revealed that the transcription level of *rinA* in the *sarA* mutant strain increased significantly compared to the wild-type (Fig. 4D). An EMSA assay was performed to establish whether SarA could regulate RinA by binding directly to the promoter. The SarA was purified in vitro to obtain higher purity proteins (Fig. 4E) and EMSA assay determined that *sarA* could directly bind to the *rinA* promoter to retard the migration of DNA probe (Fig. 4F). Taken together, these results indicated that *rinA* negatively regulates the expression of *sarA* by binding directly to the promoter and vice versa.

### Knockout of *rinA* reduces the pathogenicity of *S. aureus*

**Fig. 5:**
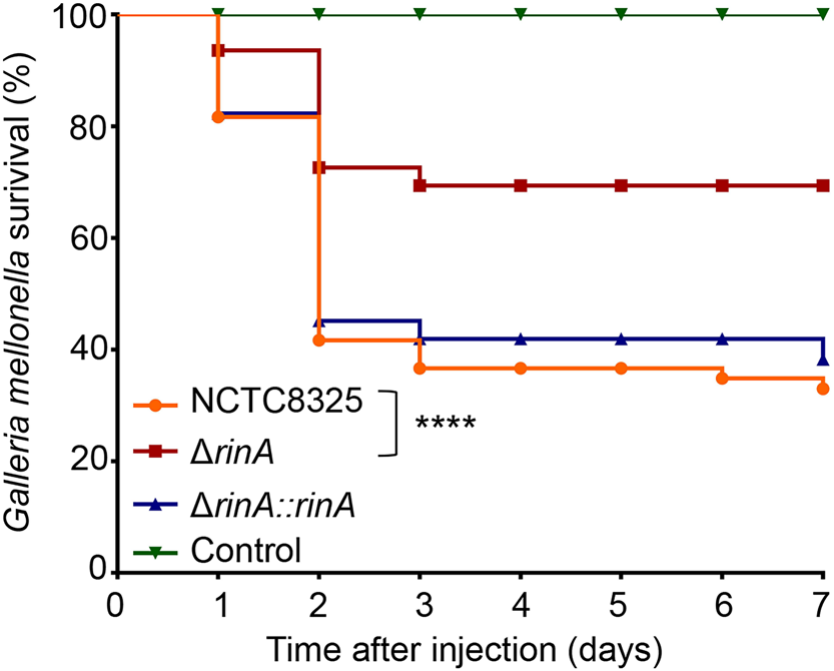
Knockout of *rinA* reduces the pathogenicity of *G.mellonella* larvae. 1 × 10^7^ CFU of the wild-type, *rinA* mutant and complementation strains were injected into *G. mellonella* larvae, PBS were used as the control group, and the survival of the *G. mellonella* larvae was recorded for 7 consecutive days after the injection. Data is presented as the mean ± standard deviations. Log-rank test is used for the comparison of statistical significance. The wild-type strain was used as the reference. ****p < 0.0001

**Fig. 6:**
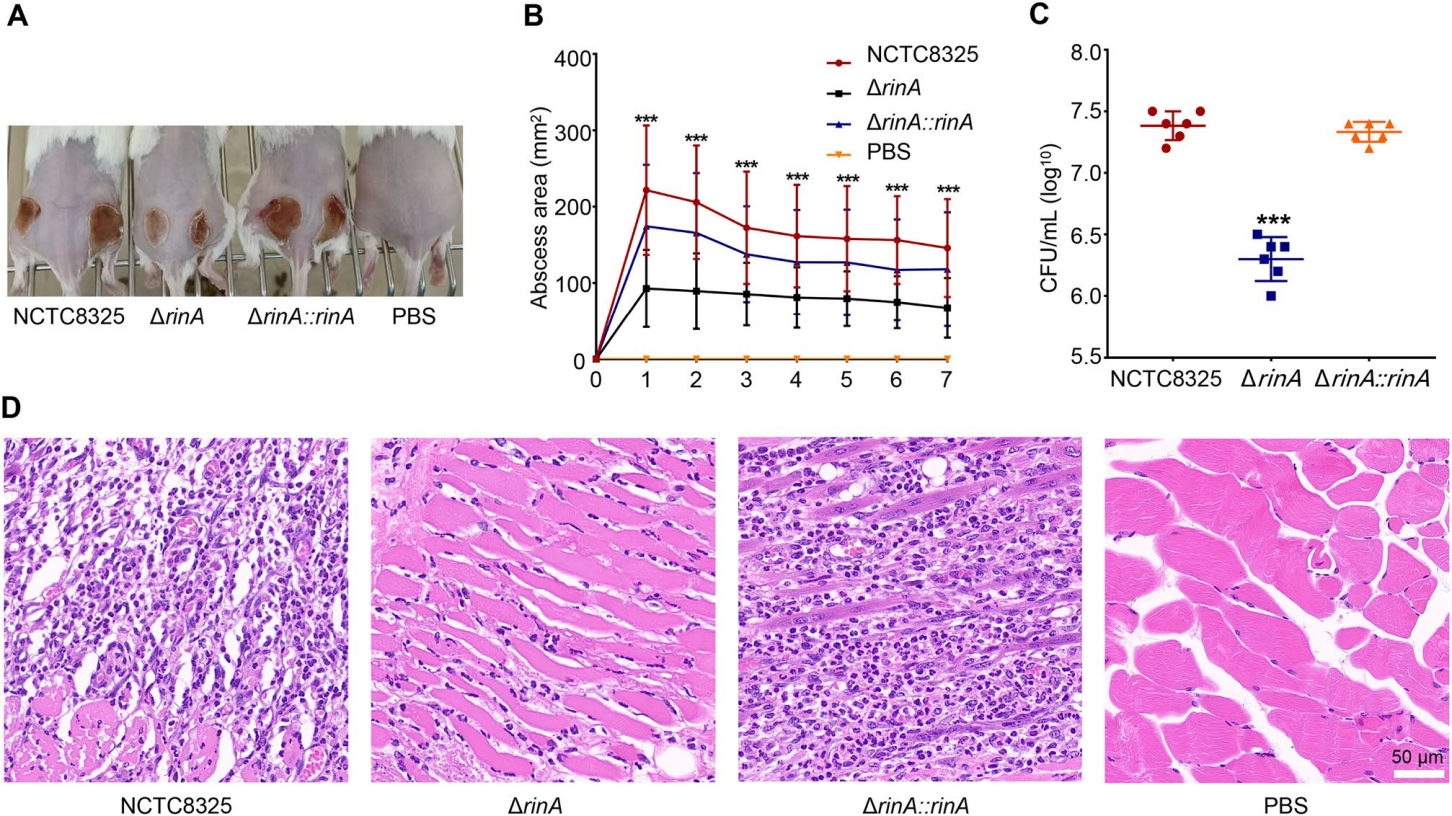
Knockout of *rinA* reduces the pathogenicity of *S. aureus* in the mouse subcutaneous abscess model. The mice were treated with 50 µL of PBS containing 5 × 10^7^ CFU of the wild-type, *rinA* mutant, and complementation strains, PBS were used as the control group. The treatment was administered in both flanks of the back by subcutaneous injection (*n* = 6–8). The abscess areas were measured daily using a caliper. **A **The photographic images of representative abscesses in mice 7 days after infection. **B** Abscess formation and skin lesion areas were monitored at 24 h intervals for 7 days. The sizes of the skin lesions were calculated by the maximal length × width. The error bars indicate the standard errors of the means, obtained from three biological replicates. Data is presented as the mean ± standard deviation. Two-tailed Student’s *t* test is used for the comparison of statistical significance. ***p < 0.001. **C** The number of CFU recovered at 7 days after infection was determined. ***p < 0.001. **D** Representative images of histological analysis (H&E staining) for samples from (**A**). The scale bar is 50 µm

We respectively selected the *G. mellonella* larvae infection and the mouse subcutaneous abscess models to verify the effects of RinA on the pathogenicity of *S. aureus*. The wild-type, *rinA* mutant and *rinA* complementation strains were inserted into the larvae, and the survival of the *G. mellonella* larvae was recorded for 7 consecutive days after the injection. The results demonstrated that compared with the *rinA* mutant strain-injected larvae, the fatality rate of *G. mellonella* larvae injected with the wild-type and complementation strains were significantly higher (Fig. 5). Next, the mouse subcutaneous abscess model was employed to test the pathogenicity, and the results revealed that compared with the wild-type and complementation strains, the subcutaneous abscess area of mice inoculated with the *rinA* mutant strain was significantly reduced (Figs. 6A, B). Then, we examined bacterial colonization of the skin lesions, and the analysis found that the number of bacterial colonization in the abscessed skins of mice inoculated with the *rinA* mutant strain was significantly lower than the wild-type (Fig. 6C). Furthermore, the histological examination of the wild-type and complementation strains exhibited more severe tissue destruction and inflammatory responses in the abscesses (Fig. 6D). Overall, these findings demonstrate that RinA plays a vital role in *S. aureus* pathogenicity.

### RinA regulates *S.aureus* virulence by governing *sarA* expression.

**Fig. 7:**
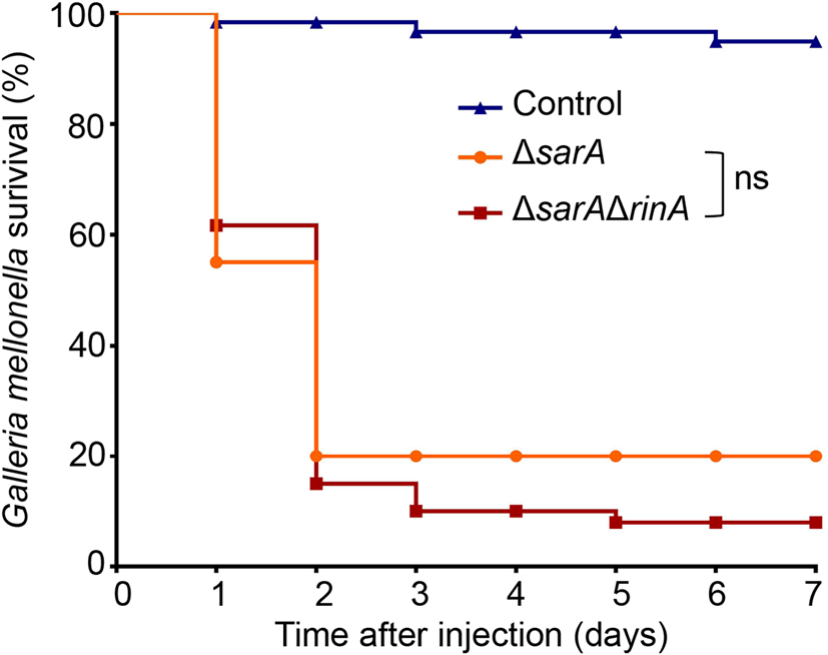
RinA regulates *S.aureus* virulence by governing *sarA* expression. 1 × 10^7^ CFU of the sar*A* mutant and *sarA rinA* double mutant strains were injected into *G. mellonella* larvae, PBS were used as the control group, and the survival of the *G. mellonella* larvae was recorded for 7 consecutive days after the injection. Data is presented as the mean ± standard deviations. Log-rank test is used for the comparison of statistical significance. The wild-type strain was used as the reference. NS, not significant (*p* > 0.05)

We selected the *G. mellonella* larvae infection to explain whether the mechanism of RinA regulates *S. aureus* virulence is related to SarA. The *sarA* mutant and *sarA rinA* double mutant strains were inserted into the larvae, and the survival of the *G. mellonella* larvae was recorded for 7 consecutive days after the injection. The results demonstrated that no significant difference was observed in the fatality rate of the *sarA* mutant and *sarA rinA* double mutant strains (Fig. 7), that means When *sarA* is knocked out, the virulence of *S.aureus* had no significantly changes whether *rinA* is knocked out or not. These results indicated that RinA regulates *S.aureus* virulence by governing *sarA* expression.

## Discussion

*S. aureus* is an opportunistic pathogen, whose colonization in humans can cause skin infections, pneumonia, bacteremia and other diseases (Horino and Hori 2020). Both antibiotic resistance and an absence of a working vaccine complicate the treatment of *S. aureus* infections (Cheung et al. 2021). Unsurprisingly, *S. aureus* infections correlate with considerable morbidity and mortality (Rasigade et al. 2014). The reason why *S. aureus* can cause serious infections is closely related to its production of virulence factors and its virulence regulatory factors. The studies on the global virulence regulators, including the Agr system, SarA family, and Sae two-component system, have shown that the virulence regulation network of *S. aureus* is highly complex (Bronner et al. 2004; Jenul and Horswill 2019).

Bacteria can acquire virulence genes through horizontal gene transfer, thus facilitating the evolution of virulence in pathogens (Dell'Annunziata et al. 2021; Juhas 2015). RinA is an important member of the RinA family that controls the transfer of virulence genes in Gram-positive bacteria. Deleting *rinA* resulted in the decrease of phage titers and significantly reduced the transfer of SAPI-encoded virulence factors, which play essential roles in bacterial evolution and pathogenesis (Ferrer et al. 2011). Together, these studies have provided a thorough understanding of RinA in the transfer of virulence genes. However, the exhaustive regulatory mechanisms mediated by RinA in virulence control are still unclear. Herein, a novel contribution of RinA in the regulation of virulence in *S. aureus* NCTC8325 was investigated.

The subcellular protein localization showed that RinA is evenly distributed in the cytoplasm of *S. aureus.* Protein subcellular localization is directly related to the disease, and proteins targeting similar subcellular localization tend to participate in mutual protein–protein interactions (PPIs) (Park et al. 2011). PPIs are vital for biological processes and are attractive pathological targets for drug discovery (Rosell and Fernandez-Recio 2018). When proteins are involved in a common biological pathway or process with disease-associated proteins, it is very plausible that they are themselves disease-associated (Barabasi et al. 2011). Hence, to understand the specific impact of RinA on the pathogenic mechanism of *S.aureus*, the subcellular localization of RinA is crucial.

Bacterial virulence is highly dynamic and context-dependent (Diard and Hardt 2017). The first metabolic dimension associated with virulence was growth. The standard virulence evolution theory assumes that virulence factors are maintained because of increasing bacterial growth within hosts and that growth influences key traits important for in vivo infection (Brown et al. 2012; Kim et al. 2021). Our results determined that the disruption of *rinA* causes growth defects in *S. aureus*. This growth defect may also be one of the reasons for the decline of pathogenicity in animal models.

Our study also illustrated how *rinA* negatively regulates the expression of *sarA* by binding directly to the promoter and vice versa. Moreover, SarA can indirectly regulate the virulence of *S. aureus* in an *agr*-dependent manner to increase the transcription levels of RNAII and RNAIII by binding to the P2 and P3 promoter regions of the *agr* locus (Cheung et al. 1997). In our experiments, the transcription levels of *agr* and *RNAIII* in wild-type and *rinA* mutant strains were not significantly different, suggesting that RinA might regulate the expression of virulence genes in an *agr*-independent manner in *S. aureus* NCTC8325. SarA can directly regulate the virulence of *S. aureus* by binding to the promoter regions of virulence factors, up-regulating virulence genes such as *hla*, *fnbA* and *tsst*, or down-regulating virulence genes such as *sspA*, *aur* and *can* (Cheung and Zhang 2002; Oscarsson et al. 2006). Since the pathogenicity of *S. aureus* was significantly reduced when *rinA* was mutated, it was speculated that the virulence regulation of *S. aureus* by RinA was achieved by modulating the transcription of *sarA* to affect the expression of the virulence genes. Interestingly, it was uncovered that RinA and SarA could regulate each other. However, this conjecture needs to be verified by further studies.

This study confirmed the critical role of RinA in *S. aureus*. It was established that the transcription level of *rinA* significantly increased in the late-logarithmic phase and that RinA was evenly distributed in the cytoplasm of *S. aureus*. Furthermore, the knockout of *rinA* significantly reduced the growth of *S. aureus*. RT-qPCR and EMSA revealed that *rinA* could negatively regulate the expression of *sarA* by directly binding to its promoter region and vice versa. The contribution of RinA to the pathogenicity of *S. aureus* was confirmed by using the infection model of *G. mellonella* larvae and the mouse model of subcutaneous abscess. These findings provide new insights into the regulatory mechanisms of virulence gene expression and the pathogenesis of *S. aureus*.

## Data Availability

All relevant data are within the manuscript and further information and requests for resources and reagents should be directed to and will be fulfilled by the corresponding author, Yujie Li (lyj2020@ustc.edu.cn).
